# Genome-wide identification and characterization of mungbean *CIRCADIAN CLOCK ASSOCIATED 1* like genes reveals an important role of *VrCCA1L26* in flowering time regulation

**DOI:** 10.1186/s12864-022-08620-7

**Published:** 2022-05-17

**Authors:** Chenyang Liu, Qianqian Zhang, Jing Dong, Chunmei Cai, Hong Zhu, Shuai Li

**Affiliations:** 1 College of Life Sciences, Key Lab of Plant Biotechnology in Universities of Shandong Province, Qingdao Agricultural University, Qingdao, 266109 China; 2 College of Agronomy, Qingdao Agricultural University, Qingdao, 266109 China

**Keywords:** Mungbean, Flowering time, *CCA1*, *VrCCA1L26*, Photoperiod

## Abstract

**Background:**

CIRCADIAN CLOCK ASSOCIATED 1 like (CCA1L) proteins are important components that participate in plant growth and development, and now have been characterized in multiple plant species. However, information on mungbean *CCA1L* genes is limited.

**Results:**

In this study, we identified 27 *VrCCA1L* genes from the mungbean genome. *VrCCA1L* genes were unevenly distributed on 10 of the 11 chromosomes and showed one tandem and two interchromosomal duplication events. Two distinct kinds of conserved MYB domains, MYB 1 and MYB 2, were found, and the conserved SHAQK(Y/F) F sequence was found at the C terminus of each MYB 2 domain. The *VrCCA1Ls* displayed a variety of exon-intron organizations, and 24 distinct motifs were found among these genes. Based on phylogenetic analysis, VrCCA1L proteins were classified into five groups; group I contained the most members, with 11 *VrCCA1Ls*. *VrCCA1L* promoters contained different types and numbers of *cis*-acting elements, and *VrCCA1Ls* showed different expression levels in different tissues. The *VrCCA1Ls* also displayed distinct expression patterns under different photoperiod conditions throughout the day in leaves. *VrCCA1L26* shared greatest homology to *Arabidopsis CCA1* and *LATE ELONGATED HYPOCOTYL* (*LHY*)*.* It delayed the flowering time in *Arabidopsis* by affecting the expression levels of *CONSTANS* (*CO*), *FLOWERING LOCUS T* (*FT*), and *SUPPRESSOR OF OVEREXPRESSION OF CONSTANS 1* (*SOC1*).

**Conclusion:**

We identified and characterized 27 *VrCCA1L* genes from mungbean genome, and investigated their spatio-temporal expression patterns. Further analysis revealed that *VrCCA1L26* delayed flowering time in transgenic *Arabidopsis* plants. Our results provide useful information for further functional characterization of the *VrCCA1L* genes.

**Supplementary Information:**

The online version contains supplementary material available at 10.1186/s12864-022-08620-7.

## Background

Mungbean [*Vigna radiata* (L.) Wilczek] is an important legume crop grown mainly in Asian countries and consumed as a common food worldwide. Mungbean seeds contain many kinds of proteins and nutrients and are used to alleviate heat shock and reduce swelling in summer [1–3]. Because of the breakdown of proteins, vitamins, and minerals, the nutritive value of mungbean seeds increases after germination [4]. Mungbean sprouts have high nutritive value and are a common vegetable food in many countries [5]. However, the production of mungbean seeds and sprouts is affected by many endogenous and environmental factors, and the investigation of functional genes based on genomic information will provide essential genetic resources for modifying mungbean plants to obtain high yield [6].

The characterization of functional genes in mungbean is limited. However, many functional genes have been identified in the past decades in multiple plant species, which provides important information for mungbean gene function analysis. Among these functional genes, transcription factors such as B-box, MADS-box, heat shock transcription factor (Hsf), and MYELOBLASTOSIS ONCOGENE (MYB) family members are important components that regulate plant growth [7–15]. Transcription factors bind to the *cis*-acting elements of their target gene promoters to regulate their expression. The MYB transcription factor family has the greatest number of members among the *Arabidopsis* gene families [16]. MYBs contain a DNA binding domain and an activation domain, and are characterized by MYB DNA binding domains at the N terminus [11]. The MYB proteins can be further grouped into five groups based on their gene structures: CIRCADIAN CLOCK ASSOCIATED 1 (CCA1)-like, CAPRICE-like, telomeric DNA binding proteins-like, I-box binding factor-like, and R-R-type proteins [7, 17]. The CCA1-like (CCA1L) proteins, which are identified based on the SHAQK(Y/F) F consensus sequence in the MYB domain, constitute the major subfamily of MYB proteins. Among these MYB proteins, CCA1L proteins have been identified to exert important functions in the control of circadian clock and flowering time, and have been identified in multiple plant species, such as *Arabidopsis*, soybean, and peach [17–20].

Among these CCA1L proteins, *CCA1* and its close homologous gene *LATE ELONGATED HYPOCOTYL* (*LHY*) have been well studied [21–24]. In *Arabidopsis*, the expression of *CCA1* or *LHY* shows a diurnal rhythm under light/dark cycle conditions and constant light or dark conditions. Overexpression of *CCA1* or *LHY* delays flowering time by regulating *FLOWERING LOCUS T* (*FT*) [25, 26]. *CCA1* and *LHY* suppresses the expression of a central circadian clock gene, *TIMING OF CAB EXPRESSION 1* (*TOC1*), by binding to the evening element (AAATATCT) in the promoter region [27]. Mutation of *CCA1* or *LHY*, disrupts circadian rhythms such as leaf movement and hypocotyl elongation [28]. Moreover, *TOC1* in turn represses the expression of *CCA1* and *LHY* from its induction at dusk until slightly before dawn [29]. Loss of function of the soybean *CCA1* and *LHY* homologs *GmLCLa1*, *GmLCLa2*, *GmLCLb1*, and *GmLCLb2* results in a short-period circadian rhythm and a late flowering phenotype [30]. In addition, some other *CCA1L* genes have been found to be involved in isoflavonoid biosynthesis, leaf senescence, seed germination, and stress response [31–35].

Although *CCA1L* genes have important roles in plant growth and development, and have been studied in many plant species [17–19], the characterization of mungbean *VrCCA1L* genes is limited. Genome-wide identification of *VrCCA1L* genes based on mungbean genomic information will provide essential information for understanding the circadian clock and flowering time regulation in mungbean [6]. In this study, we identified and characterized 27 mungbean *VrCCA1L* family members based on the conserved MYB and SHAQK(Y/F) F domains. We characterized many aspects and expression profiles of the *VrCCA1Ls*. In addition, we also investigated the function of *VrCCA1L26* in flowering time regulation. Our study provides essential information for further functional characterization of mungbean *VrCCA1L* genes.

## Methods

### Plant growth conditions

The draft genome of mungbean variety VC1973A provided by Suk-Ha Lee at Seoul National University, Seoul, South Korea, was used in this study [6]. To collect different tissues, VC1973A plants were grown in the field in Qingdao, China. Mungbean seeds were planted in the field at the end of May, and different tissues were collected when mungbean plants had produced full-length pods. Eight tissues (roots, nodule roots, shoot apices, stems, leaves, flowers, pods, and seeds) were sampled and immediately frozen in liquid nitrogen in the late afternoon (approximately 10–12 hours after dawn, approximate photoperiod conditions: 15 h light/9 h dark) in early July 2018 [36, 37]. For different photoperiod treatments, mungbean seeds were germinated in water for 1 day and then grown in soil in the growth chamber for 5 weeks. The growth conditions were set as follows: 16 h 24 °C light/8 h 24 °C dark cycles for long-day (LD) conditions, and 10 h 24 °C light/14 h 24 °C dark cycles for short-day (SD) conditions [36]. The humidity of the growth chamber was set at approximately 30%. Leaves of the mungbean plants were sampled every 4 h from lights-on at six time points under both LD and SD conditions. *Arabidopsis* plants were grown in the growth chamber under 16 h 23 °C light/8 h 21 °C dark cycle conditions. The shoots of 2-week-old *Arabidopsis* plants grown on MS agar medium were sampled every 4 h from lights-on at six time points throughout the day for gene expression analysis.

### Identification of mungbean *VrCCA1L* genes

The amino acid sequences of 20 *Arabidopsis* and 54 soybean CCA1L proteins were used as blast queries against the mungbean genome databases in Seoul National University (http://plantgenomics.snu.ac.kr/mediawiki-1.21.3/index.php/Main_Page), and National Center for Biotechnology Information (NCBI) to search for candidate genes. The presence of conserved MYB domains was verified using the Pfam database and the InterPro program [38, 39]. The presence of the SHAQK(Y/F) F motif within the MYB domain was also confirmed using blast. Predicted proteins that contained both conserved MYB and SHAQK(Y/F) F domains were designated as VrCCA1L proteins. The ProtParam program (https://web.expasy.org/protparam/) was used to predict the molecular weight (Mol. Wt) and theoretical iso-electric point (pI) of each VrCCA1L protein.

### Chromosomal locations and gene structure analyses

The physical positions of the *VrCCA1L* genes on each chromosome were obtained from NCBI and used to construct the chromosomal location map using MapInspect software (Mike Lischke, Berlin, Germany). The genomic and coding sequences (CDS) of the *VrCCA1L* genes were obtained from NCBI, and the Gene Structure Display Server program was used to analyze their gene structures [40].

### Analyses of conserved domains, conserved motifs, and sequence logos

The physical positions of the MYB domains in the VrCCA1L amino acid sequences were identified using the Pfam database and the InterPro program [38, 39]. The sequences of the conserved MYB domains were isolated from each VrCCA1L protein and used to create a sequence logo with the WebLogo platform [41]. The conserved motifs in each VrCCA1L protein were analyzed using MEME tools with default parameters [42].

### Gene duplication analysis

The OrthoMCL software was used to identify the duplicated *VrCCA1L* gene pairs as described [43, 44]. Specifically, the amino acid sequences of the VrCCA1L proteins were aligned with one another, and VrCCA1L proteins with sequence similarities greater than 70% were considered to be encoded by duplicated gene pairs.

### Analysis of *cis*-acting elements in the *VrCCA1L* promoter regions

*VrCCA1L* promoter regions 2 kb upstream of the initiation codon were obtained from NCBI and their *cis*-acting elements were analyzed using PlantCARE [45]. *Cis*-acting elements were clustered into six different types based on their potential functions as described by Hou et al. [46].

### Phylogenetic relationship analysis

The amino acid sequences of VrCAA1L*,* GmCAA1L, and AtCAA1L proteins were aligned using ClustalW2 [17, 19, 47]. The alignment result was used to construct a phylogenetic tree using MEGA 7.0, and the neighbor-joining method and default parameters were used for analysis [48]. Another phylogenetic tree was constructed with MEGA 7.0 using only VrCCA1L proteins to analyze their evolutionary relationships.

### Gene expression analysis

Total RNA was isolated from *Arabidopsis* shoots or mungbean leaves using the RNeasy mini kit (Qiagen) according to the manufacturer’s instructions. For each sample, 1 μg RNA was used for cDNA synthesis with SuperScript II reverse transcriptase (Promega). Quantitative real-time PCR (qRT–PCR) was performed as described by Li et al. [49]. Gene expression was normalized to a mungbean *Actin* gene (*Vradi03g00210*) or an *Arabidopsis Actin* (*At3g18780*) gene [50]. Three technical replicates and three biological replicates were used for each treatment to analyze gene expression. All primers used in this study are listed in Additional file 1.

### Plasmid construction and *Arabidopsis* transformation

To make a 35S:*VrCCA1L26* construct, the CDS of *VrCCA1L26* was amplified from the cDNA of VC1973A and then ligated to a modified pCAMBIA1300 vector using T4 DNA ligase (Promega) as described by Li et al. [51]. The constructed plasmid was transformed into *Arabidopsis* using the floral dip method [52]. The *VrCCA1L26* transgenic *Arabidopsis* plants were checked using PCR with specific primers, and the PCR products were sent for sequencing to confirm the sequence of the *VrCCA1L26* fragment. All primers used in this study are listed in Additional file 1.

## Results

### Identification of *VrCCA1L* genes in the mungbean genome

To identify mungbean *VrCCA1L* genes, we used the amino acid sequences of CCA1L proteins from *Arabidopsis* and soybean as blast queries against mungbean genome. Candidate genes that lacked conserved MYB and SHAQK(Y/F) F domains were discarded, and 27 *VrCCA1L* genes were confirmed in mungbean (Table 1). The *VrCCA1L* genes differed in genomic length, CDS length, and amino acid number (Table 1). The genomic length ranged from 906 (*XP_014517291* and *XP_014517292*) to 15,635 bp (*XP_014499065*), and the CDS length ranged from 594 (*XP_014499363*) to 2253 bp (*XP_014521593*). As a result, the amino acid number of the VrCCA1L proteins varied from 197 to 750. The molecular weight of VrCCA1Ls ranged from 21,774.66 (XP_014499363) to 82,272.45 Da (XP_014521593).

**Table 1:** *VrCCA1L* genes identified in mungbean genome

Gene ID	Genomic Length (bp)	CDS length (bp)	No. of AA	Mol. Wt (Da)	pI	Chr	Gene names
*XP_014511774*	4574	1434	477	52,344.16	5.32	1	*VrCCA1L1*
*XP_014503116*	7975	924	307	34,010.7	9.33	1	*VrCCA1L2*
*XP_014495340*	3123	843	280	31,439.83	9.28	3	*VrCCA1L3*
*XP_014497263*	6384	900	299	34,056.66	9.66	4	*VrCCA1L4*
*XP_014498253*	2975	909	302	32,547.43	8.94	4	*VrCCA1L5*
*XP_014499065*	15,635	2064	687	75,656.9	6.06	5	*VrCCA1L6*
*XP_014499363*	2012	594	197	21,774.66	9.7	5	*VrCCA1L7*
*XP_014502167*	4280	1062	353	38,457.15	8.95	5	*VrCCA1L8*
*XP_014502591*	3476	1263	420	46,786.58	8.37	6	*VrCCA1L9*
*XP_014505359*	2349	915	304	34,265.21	8.73	6	*VrCCA1L10*
*XP_014508244*	1148	642	213	23,941.21	10	7	*VrCCA1L11*
*XP_014510415*	1999	888	295	32,333.38	8.75	7	*VrCCA1L12*
*XP_014507110*	1599	888	295	32,234.85	6.98	7	*VrCCA1L13*
*XP_014507579*	1297	708	235	26,211.71	9.51	7	*VrCCA1L14*
*XP_014512388*	3584	918	305	33,977.52	9.04	8	*VrCCA1L15*
*XP_014512566*	2030	726	241	27,351.48	6.14	8	*VrCCA1L16*
*XP_014516756*	6136	1005	334	37,152.27	9.04	9	*VrCCA1L17*
*XP_014517291*	906	768	255	30,185.95	6.3	10	*VrCCA1L18*
*XP_014517292*	906	768	255	30,167.15	6.22	10	*VrCCA1L19*
*XP_014518898*	2789	963	320	36,181.58	9.07	10	*VrCCA1L20*
*XP_014517128*	3104	1038	345	38,087.74	8.06	10	*VrCCA1L21*
*XP_014520829*	9559	882	293	31,226.46	6.37	11	*VrCCA1L22*
*XP_014490537*	2021	912	303	32,472.26	9.67	N/A	*VrCCA1L23*
*XP_014491847*	1416	600	199	22,257.63	6.09	N/A	*VrCCA1L24*
*XP_014491921*	5543	891	296	32,540.82	9.01	N/A	*VrCCA1L25*
*XP_014521593*	9651	2253	750	82,272.45	6.04	N/A	*VrCCA1L26*
*XP_014523608*	9308	885	294	31,231.63	6.8	N/A	*VrCCA1L27*

*Chr* Chromosome number, *AA* Amino acid, *Mol. Wt* Molecular weight, *pI* Isoelectric point

### Chromosomal location and duplication analyses of *VrCCA1L* genes

Mungbean genome experienced one round of whole genome duplication during evolution and produced multiple duplicated gene pairs [6, 12]. The investigation of *VrCCA1L* chromosomal locations can provide insight into gene distributions after duplication, and a chromosomal distribution map of *VrCCA1L* genes was therefore constructed based on their physical positions (Additional file 2, Table 1). *XP_014490537*, *XP_014491847*, *XP_014491921*, *XP_014521593*, and *XP_014523608* were not included in Additional file 2 due to a lack of positional information. The *VrCCA1L* genes were designated *VrCCA1L1* to *VrCCA1L22* based on their chromosome positions (Table 1). The five *VrCCA1L* genes that lacked chromosomal position information were randomly named *VrCCA1L23* to *VrCCA1L27* (Table 1). Ten of the eleven mungbean chromosomes contained *VrCCA1L* genes, with the exception of chromosome 2. Chromosomes 7 and 10 contained the most *VrCCA1L* members, with 4 *VrCCA1L* genes on each, followed by chromosome 5, with 3 *VrCCA1L* genes (Additional file 2). *VrCCA1L18* and *VrCCA1L19* were located close together on chromosome 10.

**Fig. 1:**
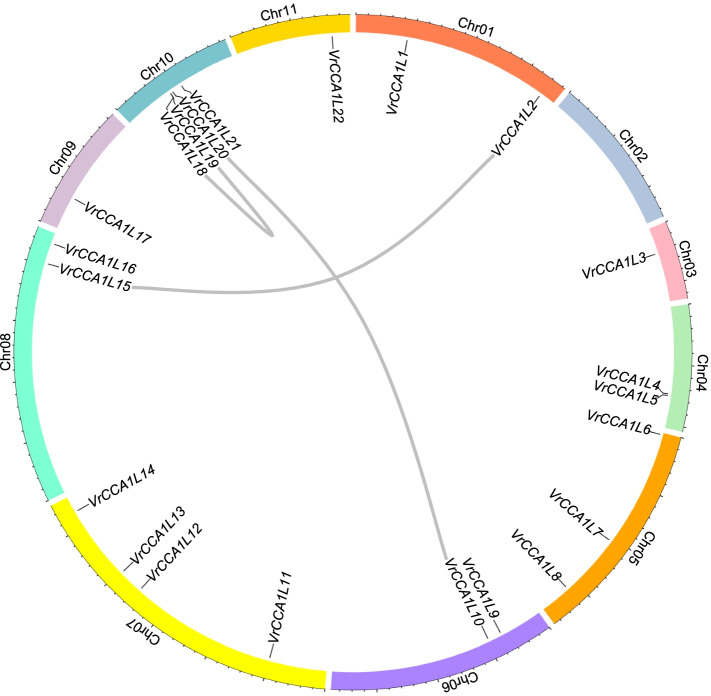
Duplication analysis of *VrCCA1L* genes. Different chromosomes are represented using different colored lines, and duplicated gene pairs are connected by grey lines. Chr indicate chromosomes

We analyzed the amino acid sequence similarities of the VrCCA1L proteins and found five duplicated gene pairs: *VrCCA1L2/VrCCA1L15*, *VrCCA1L3/VrCCA1L25*, *VrCCA1L10/VrCCA1L20*, *VrCCA1L18/VrCCA1L19*, and *VrCCA1L22/VrCCA1L27*. Three duplication events are shown in Fig. 1; *VrCCA1L3/VrCCA1L25* and *VrCCA1L22/VrCCA1L27* were not included due to a lack of positional information. The duplicated genes were located on chromosomes 1, 6, 8, and 10, and chromosome 10 contained the largest number of duplicated genes (*VrCCA1L18*, *VrCCA1L19*, and *VrCCA1L20*). *VrCCA1L2/VrCCA1L15* formed an interchromosomal duplicated gene pair, as did *VrCCA1L10/VrCCA1L20*, whereas *VrCCA1L18/VrCCA1L19* appeared to have arisen from a tandem duplication event (Fig. 1).

### Phylogenetic relationships among the *VrCCA1L* genes

**Fig. 2:**
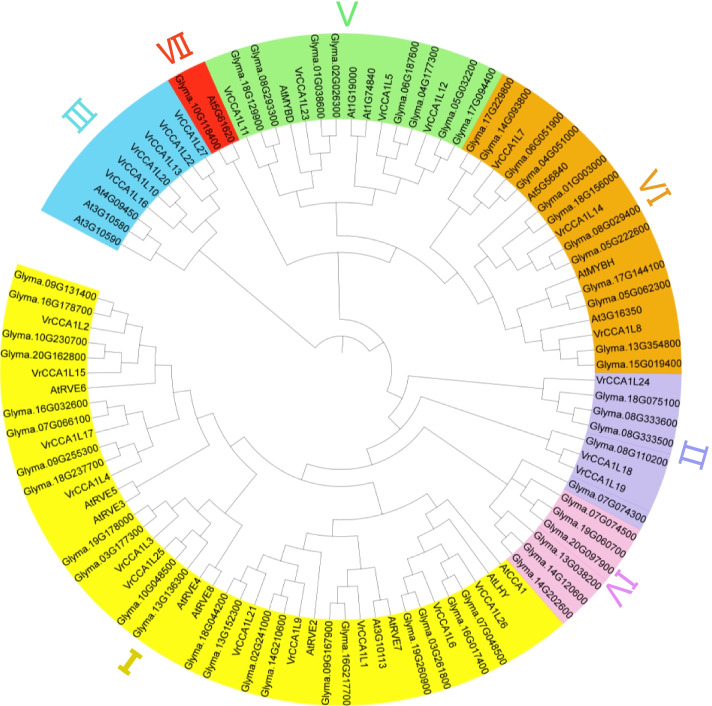
Evolutionary relationship analysis of the VrCCA1L proteins. The amino acid sequences of CCA1L proteins from *Arabidopsis*, soybean, and mungbean were used to construct a phylogenetic tree with the neighbor-joining method. Groups I to VII are presented in different colored bars. *Glyma16g01980*, *Glyma07g05410*, *Glyma03g42260*, and *Glyma19g45030* indicate *GmLCLa1*, *GmLCLa2*, *GmLCLb1*, and *GmLCLb2*, respectively

**Fig. 3:**
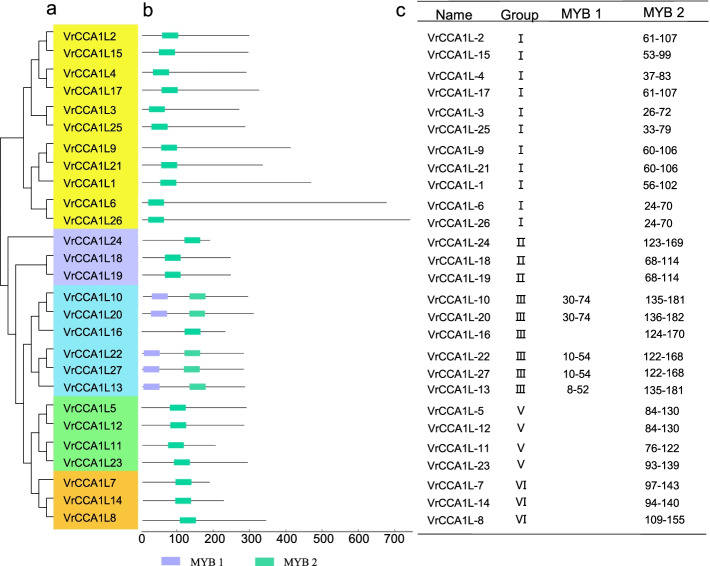
Phylogenetic relationships and conserved domains of the VrCCA1L proteins. **a** Phylogenetic analysis of the VrCCA1L proteins. Groups I to VII of VrCCA1L proteins are presented in different colored bars as described in (**b**) The positions of conserved MYB 1 and MYB 2 domains in each VrCCA1L protein. The purple and green boxes indicate MYB 1 and MYB 2 domains, respectively. **c** Classification of the VrCCA1L proteins and the positions of each MYB domain in the VrCCA1L proteins. Groups I to VII of VrCCA1L proteins, and conserved MYB 1 and MYB 2 domains are presented

To investigate the evolutionary relationships among the *VrCCA1L* genes and their homology to well-studied *CCA1L* genes from other plant species, we constructed a phylogenetic tree using 101 CCA1L proteins from *Arabidopsis*, soybean, and mungbean (Fig. 2). The *CCA1L* genes were classified into seven groups as described in soybean [19]. Group I was the largest subfamily and contained 11 *VrCCA1L* members, whereas groups IV and VII had no *VrCCA1L* members (Fig. 2). Groups II, III, V and VI contained 3, 6, 4 and 3 *VrCCA1L* members, respectively (Fig. 2). Among the *VrCCA1L* members, *VrCCA1L6* and *VrCCA1L26* showed close relationships with the well-studied *Arabidopsis* circadian clock and flowering time genes *CCA1* and *LHY*, as well as the soybean genes *GmLCLa1* (*Glyma16g01980*), *GmLCLa2* (*Glyma07g05410*), *GmLCLb1* (*Glyma03g42260*), and *GmLCLb2* (*Glyma19g45030)* [30]. These results suggest that *VrCCA1L6* and *VrCCA1L26* may be key factors involved in circadian clock and flowering time regulation in mungbean. We also constructed a phylogenetic tree using only *VrCCA1L* genes and found that all genes from the same group were clustered together (Fig. 3a). Moreover, the genes from each pair of duplicated genes were placed into the same clades in the phylogenetic tree (Fig. 3a).

### Analysis of VrCCA1L conserved domains

**Fig. 4:**
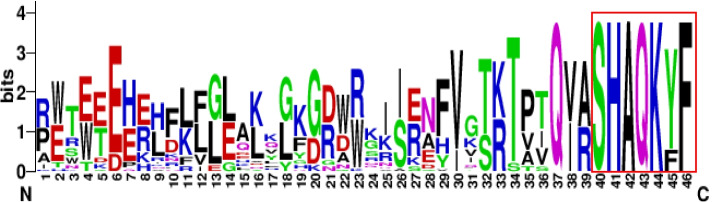
Sequence logo of the conserved MYB 2 domain. The red box at the C terminus indicates the conserved SHAQK(Y/F) F domain

The CCA1L protein is a member of the MYB gene family and contains MYB domains [7, 17]. Different CCA1L proteins contain different numbers and types of conserved MYB domains [17–20], and we therefore analyzed the MYB domain numbers and types in the VrCCA1L proteins. Two distinct kinds of conserved MYB domain, MYB 1 and MYB 2, were found (Fig. 3b, Additional file 3). All members of groups I, II, V, and VI contained only one MYB 2 domain. By contrast, most group III members contained one MYB 1 and one MYB 2 domain, with the exception of VrCCA1L16, which had only one MYB 2 domain (Fig. 3b, c). All the MYB 1 domains were found close to the N termini of the VrCCA1L proteins (Fig. 3b, c). In addition, the conserved SHAQK(Y/F) F sequence of the VrCCA1L proteins was found at the C termini of the MYB 2 domains (Fig. 4, Additional file 3).

### Exon-intron organization and conserved motif analyses of the *VrCCA1L* genes

**Fig. 5:**
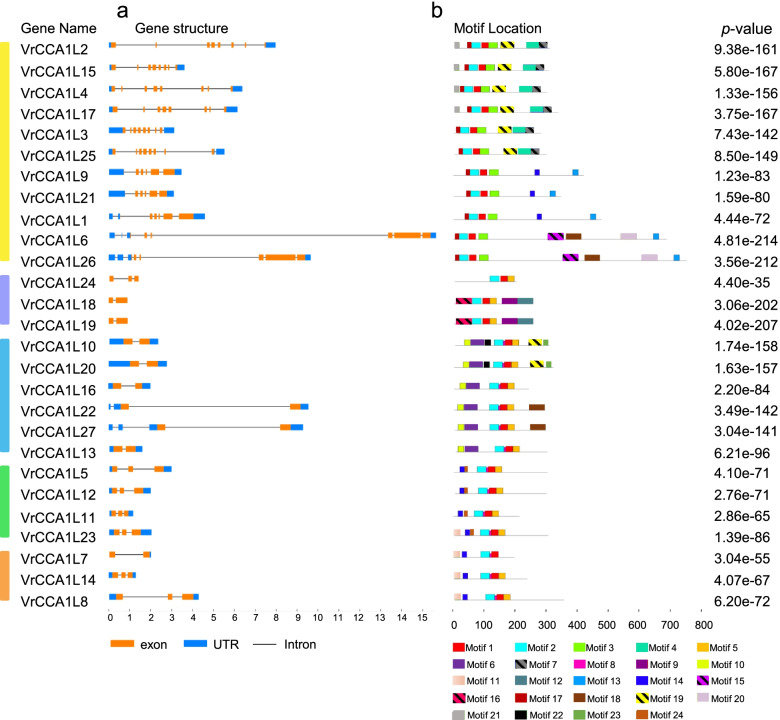
Gene structures and conserved motifs of the *VrCCA1L* genes. **a** Exon-intron organization of the *VrCCA1L* genes. The orange, blue, and black boxes indicate exons, UTRs, and introns, respectively. (b) Conserved motifs in the VrCCA1L proteins. Motifs 1–24 are presented in different colored boxes

To investigate the similarity and diversity in gene structures of the *VrCCA1L* genes, we analyzed their full-length genomic and CDS using the Gene Structure Display Server program [40]. Twenty-four of the 27 *VrCCA1L* genes contained UTRs, with the exception of all group II members (Fig. 5a). All group I members contained a relatively large number of exons, ranging from 5 to 8 (Fig. 5a). Most group II and III genes had 2 exons, with the exception of *VrCCA1L24*, which had 3 exons. All group V *VrCCA1Ls* had 3 exons, and 1 and 2 members of group VI contained 2 and 3 exons, respectively. To further analyze the conservation and diversity of the *VrCCA1L* gene structures, we analyzed conserved motifs in their encoded proteins using MEME tools [42]. Twenty-four distinct motifs were identified (Fig. 5b, Additional file 4). All VrCCA1L proteins contained motif 2, which appeared to represent the conserved MYB 2 domain. Most members within a group shared some motifs; for example, group V members contained motifs 1, 2, 5, 10, and 11 (Fig. 5b), indicating that these genes may share some common gene structures. Other motifs were found only in specific groups; for example, motif 4 was found only in group I, and motif 10 was found only in group III (Fig. 5b), highlighting the structure diversity of VrCCA1L proteins from different groups.

### *Cis*-acting elements in the *VrCCA1L* promoter regions

To predict the *cis*-acting elements in the *VrCCA1L* promoter regions, 2 kb of sequence upstream of the ATG initiation codon for each gene was downloaded from NCBI and analyzed using PlantCare [45]. Eighty-seven *cis*-acting elements were obtained across all the *VrCCA1L* promoters, and 56 were predicted to have potential functions (Additional file 5). The *VrCCA1L* genes contained various numbers and types of *cis*-acting elements, again highlighting their functional diversity (Additional file 5). Based on their predicted functions, the *cis*-acting elements were classified into six groups as described by Jin et al. [44]: development-related elements, environmental stress-related elements, hormone-responsive elements, light-responsive elements, promoter-related elements, and site binding-related elements (Table 2). All *VrCCA1L* genes contained light-responsive elements and promoter-related elements. The light-responsive element was the most abundant element in each *VrCCA1L* promoter, and the number of light-responsive elements ranged from 3 (*VrCCA1L24*) to 11 (*VrCCA1L13*) (Table 2). The promoter-related elements CAAT-box and TATA-box, which are basic promoter elements, and the light-responsive element Box 4 were found in all *VrCCA1L* promoters (Additional file 5). The number of *cis*-acting elements in each duplicated gene pair varied, indicating that these duplicated genes may exhibit distinct expression responses under specific conditions (Table 2).

**Table 2:** Numbers and types of *cis*-acting elements in each *VrCCA1L* promoter region

Gene name	Development related elements	Environmental stress related elements	Hormone-responsive elements	Light-responsive elements	Promoter related elements	Site-binding related elements	Others
*VrCCA1L1*	0	1	2	9	2	0	9
*VrCCA1L2*	1	1	3	7	2	0	10
*VrCCA1L3*	3	4	1	5	2	0	8
*VrCCA1L4*	0	2	4	10	2	0	9
*VrCCA1L5*	2	1	2	5	2	0	12
*VrCCA1L6*	3	3	5	9	2	1	13
*VrCCA1L7*	3	2	4	5	2	0	9
*VrCCA1L8*	2	0	1	9	2	0	14
*VrCCA1L9*	2	3	4	7	2	0	14
*VrCCA1L10*	1	2	2	6	2	0	11
*VrCCA1L11*	0	3	4	9	3	1	11
*VrCCA1L12*	1	2	2	8	3	0	12
*VrCCA1L13*	0	1	1	11	2	1	14
*VrCCA1L14*	1	2	4	8	2	0	12
*VrCCA1L15*	2	1	3	8	2	0	17
*VrCCA1L16*	1	3	4	8	2	0	14
*VrCCA1L17*	3	0	3	6	2	2	12
*VrCCA1L18*	1	0	3	6	2	0	8
*VrCCA1L19*	1	0	3	8	2	0	7
*VrCCA1L20*	0	1	5	6	2	0	14
*VrCCA1L21*	2	4	4	7	2	2	15
*VrCCA1L22*	1	3	3	8	2	2	10
*VrCCA1L23*	1	1	4	6	2	0	13
*VrCCA1L24*	0	0	0	3	2	0	5
*VrCCA1L25*	3	1	4	6	2	2	14
*VrCCA1L26*	3	3	4	5	2	1	10
*VrCCA1L27*	0	2	5	9	2	1	9

### Expression of *VrCCA1L* genes in different tissues

**Fig. 6:**
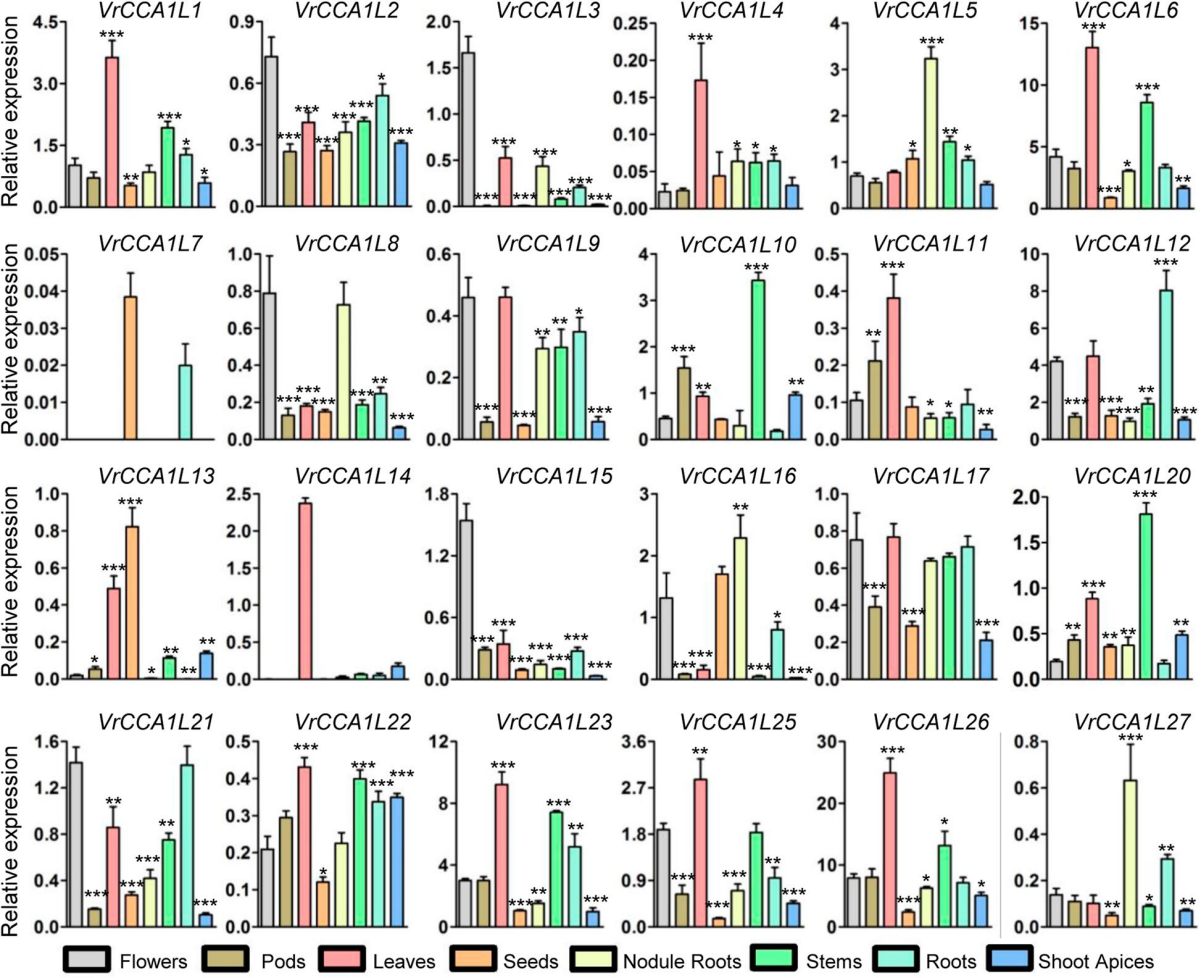
Expression analyses of the *VrCCA1L* genes in different tissues. The expression patterns of *VrCCA1L* genes were analyzed using qRT–PCR. All the *VrCCA1L* genes were firstly normalized to a mungbean *Actin* gene (*Vradi03g00210*). Then the expression level of *VrCCA1L1* in flowers was set to 1, and the values of all the *VrCCA1L* genes in different tissues were adjusted accordingly. Significant differences relative to the expression in flowers are indicated by asterisks (***P < 0.001; **P < 0.01; and *P < 0.05)

To address the potential functions of *VrCCA1L* genes, eight tissues were sampled from the reference genome variety VC1973A at a single time point and used for gene expression analysis: roots, nodule roots, shoot apices, stems, leaves, flowers, pods, and seeds [36, 37]. *VrCCA1L* genes showed distinct expression levels in different tissues at the tested time point. For example, *VrCCA1L12* showed relatively high expression levels in most tissues examined (expression level > 1) (Fig. 6). By contrast, *VrCCA1L18*, *VrCCA1L19*, and *VrCCA1L24* could not be detected in any tissues, indicating that their transcript abundances were extremely low (data not shown). Some *VrCCA1L* genes were expressed at high abundance only in specific tissues at the tested time point. For example, *VrCCA1L14* showed high expression levels in leaves but low levels elsewhere, indicating that it may have a critical function in leaves. In addition, the expression of orthologs of the well-studied circadian clock genes *CCA1* and *LHY* (*VrCCA1L6* and *VrCCA1L26*) was also analyzed at the tested time point. *VrCCA1L26* was expressed at relatively high levels in all tissues; *VrCCA1L6* showed relatively high abundance in most tissues, with the exception of seeds (Fig. 6).

Duplicated genes may retain some common functions from the original gene and may also acquire new functions during evolution [53]. We therefore analyzed the expression of duplicated *VrCCA1L* genes in multiple tissues at the tested time point. Some duplicated pairs showed similar expression patterns in some tissues but distinct expression levels in others (Fig. 6). For example, the tandem duplicates *VrCCA1L18* and *VrCCA1L19* showed extremely low expression levels in all the tissues examined at the tested time point, suggesting that these two genes may have similar transcriptional regulatory mechanisms. By contrast, *VrCCA1L10* and *VrCCA1L20* exhibited similar expression levels in leaves and roots but different expression patterns in flowers, stems, pods, seeds, nodule roots, and shoot apices (Fig. 6), indicating that they acquired different transcriptional regulatory mechanisms in these tissues after genome duplication.

### Expression patterns of *VrCCA1Ls* under different photoperiod conditions in leaves

**Fig. 7:**
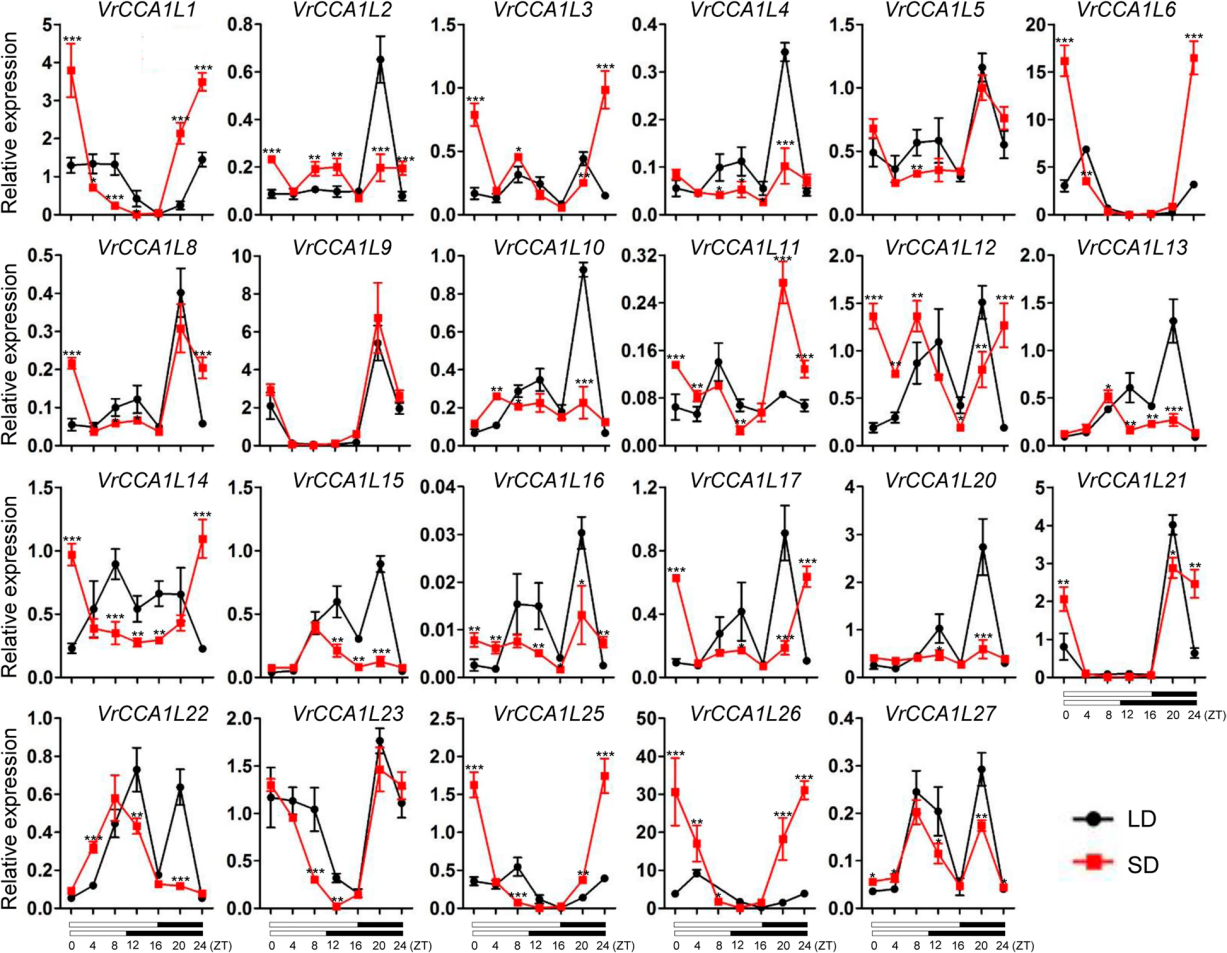
Expression analysis of the *VrCCA1L* genes throughout the day under LD and SD conditions. The photoperiod treatments were 16 h light/8 h dark and 10 h light/14 h dark cycles for LD and SD conditions, respectively. ZT, Zeitgeber Time. The *x*-axis indicates the light and dark cycle throughout the day. Open boxes indicate the time of lights-on, and black boxes indicate the time of darkness. Red lines indicate SD and black lines mean LD. Expression levels of the *VrCCA1Ls* were normalized to that of an *Actin* gene from mungbean. Significant differences relative to the expression under LD conditions are indicated with asterisks (***P < 0.001; **P < 0.01; and *P < 0.05)

Because the expression of *Arabidopsis CCA1* and *LHY* shows different expression levels under different photoperiod conditions, we investigated the expression patterns of *VrCCA1L* genes in mungbean leaves during the day and night under LD and SD conditions (Fig. 7). *VrCCA1L7*, *VrCCA1L18*, *VrCCA1L19*, and *VrCCA1L24* were not detected in leaves during the day under either LD or SD conditions. Most *VrCCA1L* genes showed different expression levels in leaves under LD and SD conditions, with the exception of *VrCCA1L9*, whose expression was not regulated by day length (Fig. 7), indicating that *VrCCA1L* genes are important factors in response to different photoperiod conditions. Several *VrCCA1L* genes might be diurnal rhythm genes in leaves under both LD and SD conditions. For example, the expression of *VrCCA1L23* decreased during the day and increased during the night under both LD and SD conditions (Fig. 7). By contrast, some *VrCCA1L* genes might be diurnal rhythm genes in leaves only under specific day length conditions. For example, *VrCCA1L2* exhibited increased expression during the night, and decreased during the day in leaves under LD conditions but not SD conditions, indicating that these genes may be involved in photoperiod-dependent regulatory pathways. Some duplicated genes showed similar expression patterns in leaves under specific photoperiod conditions. For example, *VrCCA1L10* and *VrCCA1L20* showed similar expression patterns in leaves under LD conditions. By contrast, some duplicated gene pairs, such as *VrCCA1L2* and *VrCCA1L15*, exhibited distinct expression patterns in leaves under LD and SD conditions, indicating functional differentiation of these duplicated genes (Fig. 7).

### *VrCCA1L26* influenced flowering time in *Arabidopsis*

**Fig. 8:**
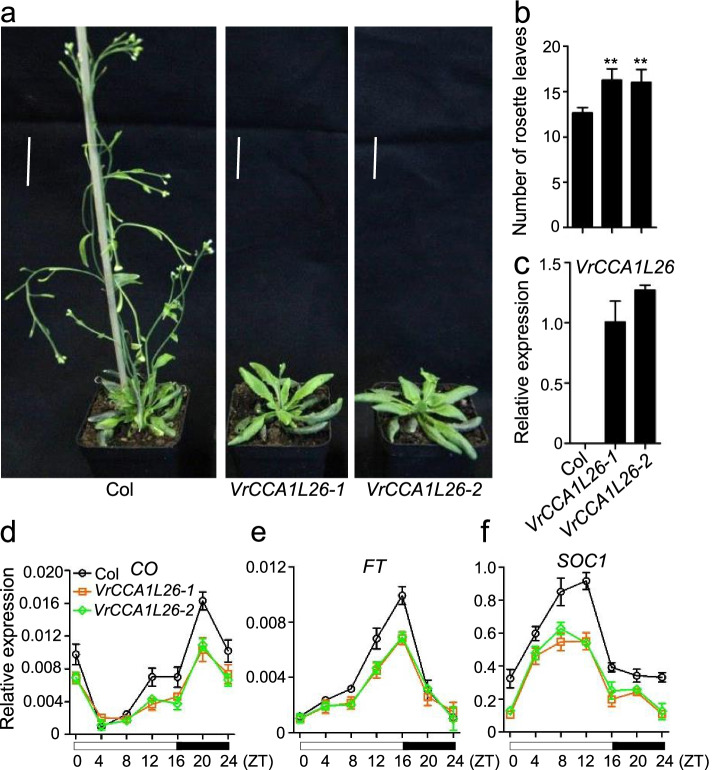
Flowering time phenotypes of the *VrCCA1L26* transgenic *Arabidopsis* plants. **a** Flowering time of *VrCCA1L26* transgenic and wild-type *Arabidopsis* plants. *Arabidopsis* plants were grown in the growth chamber under 16 h 23 °C light/8 h 21 °C dark cycle conditions. Bars = 4 cm. **b** The number of rosette leaves in *VrCCA1L26* transgenic and wild-type plants. The rosette leaf numbers of *VrCCA1L26* transgenic and wild-type plants were recorded after the bolting of *Arabidopsis* plants. **c** The expression levels of *VrCCA1L26* in *VrCCA1L26* transgenic and wild-type plants. The expression level of *VrCCA1L26* in transgenic line 1 was set to 1, and the values for other transgenic and wild-type plants were adjusted accordingly. Significant differences relative to the corresponding wild-type plants are indicated with asterisks (**P < 0.01). **d**-**f** The expression levels of *CO* (d), *FT* (**e**), and *SOC1* (**f**) in *VrCCA1L26* transgenic and wild-type plants. The *x*-axis indicates the light and dark cycle throughout the day. The expression levels of *CO*, *FT*, and *SOC1* were normalized to *AtActin*. Open boxes indicate the time of lights-on, and black boxes indicate the time of darkness. ZT, Zeitgeber Time (ZT)

*VrCCA1L6* and *VrCCA1L26* showed a close phylogenetic relationship with *Arabidopsis CCA1* and *LHY*, and the expression levels of *VrCCA1L26* were higher than those of *VrCCA1L6* in most tested tissues. We therefore selected *VrCCA1L26* for functional characterization in this study, and we transformed *VrCCA1L26* into *Arabidopsis* for further analysis (Additional file 6). *VrCCA1L26* exhibited high expression levels in the transgenic lines, whereas *VrCCA1L26* was not detected in wild-type *Arabidopsis* plants (Fig. 8). *VrCCA1L26* transgenic lines showed greater numbers of rosette leaves than wild-type *Arabidopsis* plants (Fig. 8), indicating that *VrCCA1L26* delayed flowering time in *Arabidopsis*. To search for the factors responsible for the delayed flowering time in *VrCCA1L26* transgenic plants, we investigated the expression of several flowering time–related genes, including *CO*, *FT*, and *SOC1*, all of which accelerate flowering in *Arabidopsis* [54, 55]. The expression levels of *CO*, *FT*, and *SOC1* were reduced in *VrCCA1L26* transgenic *Arabidopsis* lines at several time point throughout the day, indicating that *VrCCA1L26* may regulate flowering time by affecting expression of these flowering time genes (Fig. 8).

## Discussion

*CCA1L* transcription factors, especially the circadian clock and flowering time regulation genes *CCA1* and *LHY*, play critical roles in plant growth and development and have been identified in several plant species [17–19]. Investigation of *VrCCA1L* genes in mungbean will increase our understanding of its growth and development. In this study, we identified 27 mungbean *VrCCA1L* genes and investigated many of their characteristics.

The number of *CCA1L* genes varies among different plant species. Of the seven *CCA1L* groups, group I has the largest number of *CCA1L* genes in soybean, mungbean, and *Arabidopsis* (Fig. 2), indicating the evolutionary conservation of *CCA1L* genes in different plant species. Most of the seven groups are shared among multiple plant species, but some groups have been lost during evolution. For example, mungbean has no group IV or VII members, and soybean has no group III *GmCCA1L* members, indicating functional divergence of *CCA1L* genes in different plant species (Fig. 2). Moreover, the number of *CCA1Ls* in mungbean is half that in soybean. This reflects the fact that soybean has experienced two rounds of whole genome duplication [56], whereas mungbean has experienced only one [6]. However, the numbers of *VrCCA1L* genes in most of the seven groups in mungbean are not half of those in soybean, with the exception of group I (11 members in mungbean, 22 in soybean), indicating that the gene structures of many CCA1L proteins in legumes have changed during evolution.

*Cis*-acting elements are essential factors for gene expression, and several circadian clock related *cis*-acting elements have been found in *VrCCA1L* promoters (Additional file 5). For example, the G-box *cis*-acting element is necessary for the transcriptional regulation of *CCA1* in *Arabidopsis* [57], and have been found in many *VrCCA1L* promoters, including *VrCCA1L6* and *VrCCA1L26*, indicating that these *VrCCA1L* genes play important roles in circadian clock regulation. The expression of *VrCCA1L* genes in different tissues provides insight into their potential functions. The numbers and types of *cis*-acting elements varied among the promoter regions of different *VrCCA1L* genes (Table 2), and this may be responsible for their different expression patterns in various tissues at the tested time point and under contrasting photoperiod conditions in leaves (Figs. 6 and 7). Although all the *VrCCA1L* promoters contained *cis*-acting elements, the expression of several *VrCCA1L* members could not be detected in any tissues examined at the tested time point; these included *VrCCA1L18*, *VrCCA1L19*, and *VrCCA1L24*. The expression of many *CCA1L* genes is controlled by the circadian clock and changes during the day and night in leaves. For example, the expression of *CCA1* and *LHY* shows a diurnal rhythm [21, 22, 28]. However, the expression of *VrCCA1L18*, *VrCCA1L19*, and *VrCCA1L24* was not detected throughout the day under either LD or SD conditions in leaves. Many factors affect gene expression in addition to *cis*-acting elements. For example, epigenetic modifications such as DNA methylation have a substantial effect on gene expression [58, 59]. The extremely low expression of *VrCCA1L18*, *VrCCA1L19*, and *VrCCA1L24* in all tissues at the tested time point and under all photoperiod conditions in leaves may therefore reflect epigenetic modification. These genes may be expressed at high levels under other growth conditions.

Mungbean genome has experienced one round of whole genome duplication, which may have produced many novel genes [6, 12, 53]. Five duplicated gene pairs were found among the 27 *VrCCA1L* genes (Fig. 1). Duplicated genes have evolved from a single ancestor and may retain some common functions. For example, the duplicated genes *VrCCA1L18/VrCCA1L19* have similar exon-intron structures and conserved motifs (Fig. 5). Moreover, *VrCCA1L18* and *VrCCA1L19* both showed extremely low expression in different tissues at the tested time point and under different photoperiod conditions in leaves, suggesting that they may share some common functions. By contrast, some duplicated genes appeared to have obtained new functions and showed functional diversity. For example, *VrCCA1L10* and *VrCCA1L20* had different expression levels in several tissues at the tested time point, indicating different potential functions (Fig. 6). In addition, the different motifs in these VrCCA1L proteins indicate structure diversity of *VrCCA1L* genes. Some *VrCCA1L* genes shared some common motifs, such as motif 2, indicating that these motifs might be historical structures among these genes (Fig. 5). Some motifs were found in some specific groups, suggesting that the*VrCCA1L* genes in these groups obtained new gene structures during evolution.

In *Arabidopsis*, *CCA1* and *LHY* are critical components involved in circadian clock and flowering time regulation [21, 22]. Loss of function of the soybean homologs *GmLCLa1*, *GmLCLa2*, *GmLCLb1*, and *GmLCLb2* results in a short-period circadian rhythm and a late flowering phenotype [30]. The expression of many *VrCCA1L* genes was regulated by photoperiod and showed different expression levels during the day and night under either LD or SD conditions in leaves (Fig. 7). Moreover, most of the *VrCCA1L* genes showed different expression patterns under LD and SD conditions throughout the day in leaves (Fig. 7), indicating that they may have distinct roles under different photoperiod conditions. Whether *VrCCA1L* genes are diurnal rhythm factors still need further investigation under a full forty-eight-hour time course and constant (free-running) conditions. *VrCCA1L26* showed a close relationship with *Arabidopsis CCA1* and *LHY*, as well as soybean *GmLCLa1*, *GmLCLa2*, *GmLCLb1*, and *GmLCLb2* (Fig. 2). It also showed relatively high expression levels in most tissues at the tested time point (Fig. 6). When expressed in transgenic *Arabidopsis*, it delayed flowering time by suppressing *CO*, *FT*, and *SOC1* expression (Fig. 8). *VrCCA1L26* exhibited distinct expression patterns under different photoperiod conditions in leaves, but whether it has different functions in flowering time regulation under different photoperiod conditions requires further investigation. *Arabidopsis CCA1* and *LHY* delay flowering time, and *VrCCA1L26* also delayed flowering time in transgenic *Arabidopsis* plants. By contrast, soybean *GmLCLa1*, *GmLCLa2*, *GmLCLb1*, and *GmLCLb2* are essential for accelerating flowering in soybean [21, 22, 30]. *CCA1* and *LHY* homologs therefore have distinct functions in flowering time regulation in different plant species. Our study provides evidence that *VrCCA1L26* suppresses flowering in *Arabidopsis*, but we do not yet know its function in mungbean. Whether *VrCCA1L26* has similar functions in regulation of flowering time in mungbean and *Arabidopsis* will require further investigation. *Arabidopsis CCA1* and *LHY* are also involved in the regulation of hypocotyl elongation [21, 22, 28], and *VrCCA1L26* overexpression plants showed elongated hypocotyl in *Arabidopsis* (Additional file 7), thus *VrCCA1L26* may be a key factor that influences mungbean sprout production. In addition, *VrCCA1L26* may interact with *LHY* or *CCA1* to regulate the expression of circadian clock genes in *Arabidopsis*, thereby influencing circadian clock regulation. The mechanisms by which *VrCCA1L26* affects circadian clock regulation require further investigation.

## Conclusion

In summary, we identified 27 *VrCCA1L* genes from mungbean and investigated many of their characteristics, such as chromosomal locations, exon-intron organization, conserved domains, conserved motifs, *cis*-acting elements, duplicated gene pairs, and expression patterns in different tissues at a single time and under different photoperiod conditions in leaves. In addition, our analysis revealed that *VrCCA1L26* delayed flowering time in transgenic *Arabidopsis* plants. Our results provide essential information for further functional characterization of *VrCCA1L* genes and circadian clock regulation in mungbean.

## Supplementary Information

**Additional file 1.**

**Additional file 2.**

**Additional file 3.**

**Additional file 4.**

**Additional file 5.**

**Additional file 6.**

**Additional file 7.**

## Data Availability

All data used in this study are included in this article and additional files. The genome sequence and annotation datasets that supported our findings are available in: *Arabidopsis* (http://www.arabidopsis.org), soybean (https://soybase.org/) and mungbean (http://plantgenomics.snu.ac.kr/mediawiki-1.21.3/index.php/Main_Page) (https://www.ncbi.nlm.nih.gov/). All the mungbean genes used in this study for phylogeny and subsequent analysis are mentioned in Table 1 and can be downloaded from NCBI.

## Abbreviations

Vr: Vigna radiate
LD: long-day
SD: Short-day
LHY: LATE ELONGATED HYPOCOTYL
CO: CONSTANS
FT: FLOWERING LOCUS T
SOC1: SUPPRESSOR OF OVEREXPRESSION OF CONSTANS 1
CCA1: CIRCADIAN CLOCK ASSOCIATED 1
Hsf: Heat Shock transcription Factor
MYB: MYELOBLASTOSIS ONCOGENE
TOC1: TIMING OF CAB EXPRESSION 1
ZT: Zeitgeber Time
CDS: Coding sequences
