# Supplementary material for: Genome-wide identification and characterization of mungbean CIRCADIAN CLOCK ASSOCIATED 1 like genes reveals an important role of VrCCA1L26 in flowering time regulation
Source: BMC Genomics. 2022 May 17;23:374. doi: 10.1186/s12864-022-08620-7 (PMC9115955; doi:10.1186/s12864-022-08620-7)
Supplement: Supplementary file 2 — Additional file 2. [file 12864_2022_8620_MOESM2_ESM.pptx]

## Slide 1
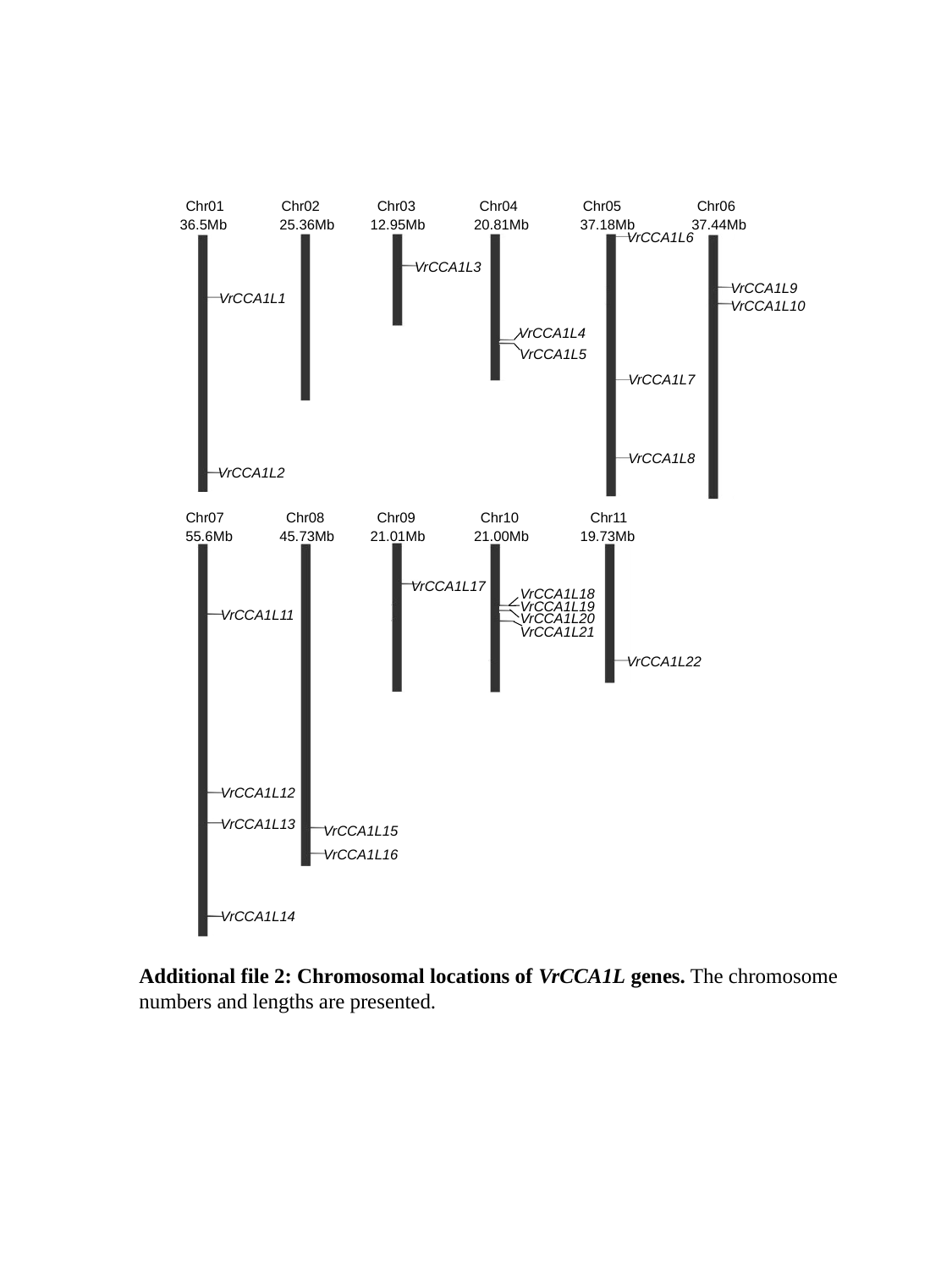

Chr01
Chr02
Chr03
Chr04
Chr05
Chr06
36.5Mb
25.36Mb
12.95Mb
20.81Mb
37.18Mb
37.44Mb
VrCCA1L6
VrCCA1L3
VrCCA1L9
VrCCA1L1
VrCCA1L10
VrCCA1L4
VrCCA1L5
VrCCA1L7
VrCCA1L8
VrCCA1L2
Chr07
Chr08
Chr09
Chr10
Chr11
55.6Mb
45.73Mb
21.01Mb
21.00Mb
19.73Mb
VrCCA1L17
VrCCA1L18
VrCCA1L19
VrCCA1L11
VrCCA1L20
VrCCA1L21
VrCCA1L22
VrCCA1L12
VrCCA1L13
VrCCA1L15
VrCCA1L16
VrCCA1L14
Additional file 2: Chromosomal locations of VrCCA1L genes. The chromosome numbers and lengths are presented.
