# Supplementary material for: Genome-wide identification and characterization of mungbean CIRCADIAN CLOCK ASSOCIATED 1 like genes reveals an important role of VrCCA1L26 in flowering time regulation
Source: BMC Genomics. 2022 May 17;23:374. doi: 10.1186/s12864-022-08620-7 (PMC9115955; doi:10.1186/s12864-022-08620-7)
Supplement: Supplementary file 3 — Additional file 3. [file 12864_2022_8620_MOESM3_ESM.pptx]

## Slide 1
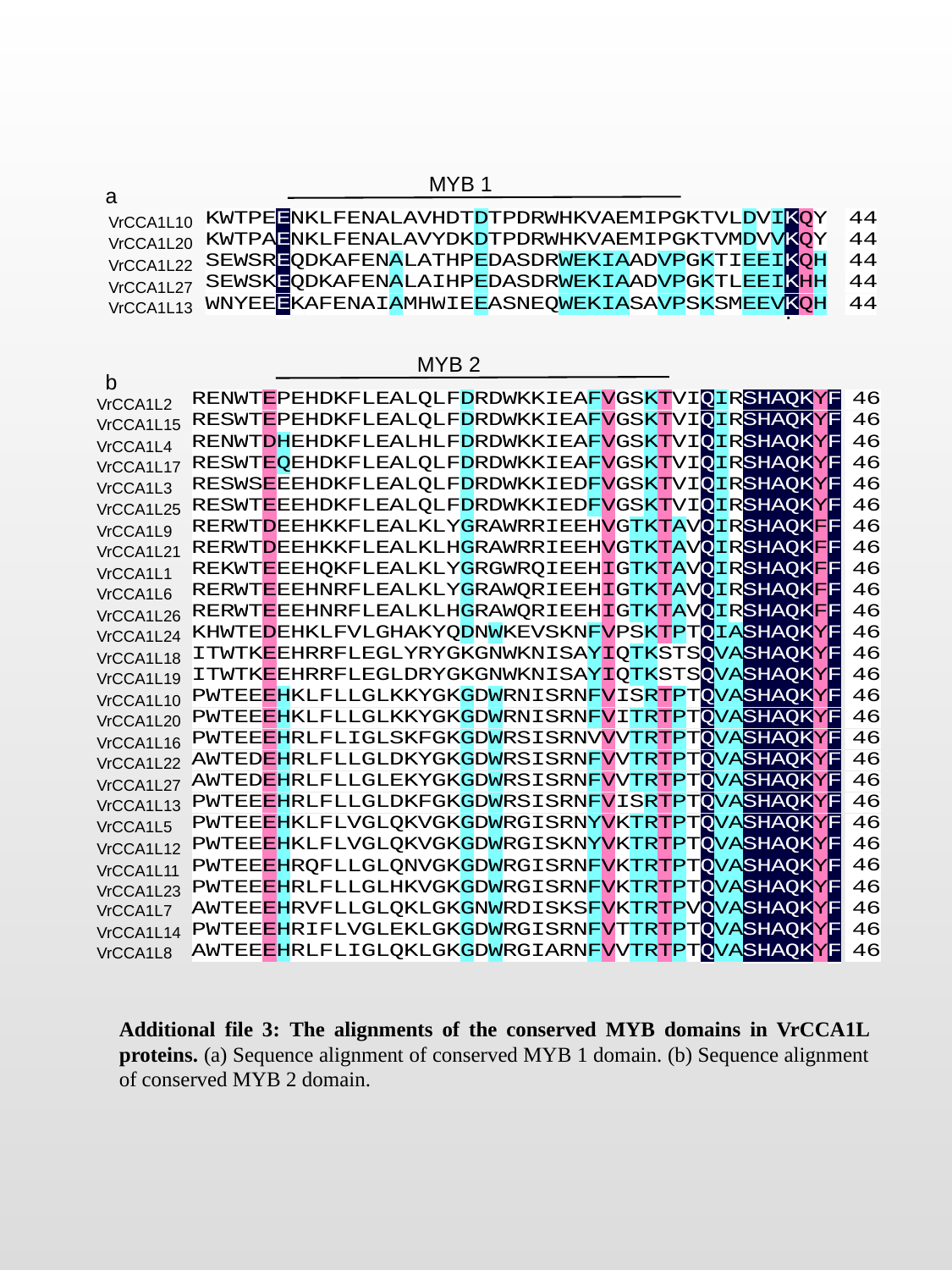

MYB 1
VrCCA1L10
VrCCA1L20
VrCCA1L22
VrCCA1L27
VrCCA1L13
a
MYB 2
VrCCA1L2
VrCCA1L15
VrCCA1L4
VrCCA1L17
VrCCA1L3
VrCCA1L25
VrCCA1L9
VrCCA1L21
VrCCA1L1
VrCCA1L6
VrCCA1L26
VrCCA1L24
VrCCA1L18
VrCCA1L19
VrCCA1L10
VrCCA1L20
VrCCA1L16
VrCCA1L22
VrCCA1L27
VrCCA1L13
VrCCA1L5
VrCCA1L12
VrCCA1L11
VrCCA1L23
VrCCA1L7
VrCCA1L14
VrCCA1L8
b
Additional file 3: The alignments of the conserved MYB domains in VrCCA1L proteins. (a) Sequence alignment of conserved MYB 1 domain. (b) Sequence alignment of conserved MYB 2 domain.
