# Supplementary material for: Genome-wide identification and characterization of mungbean CIRCADIAN CLOCK ASSOCIATED 1 like genes reveals an important role of VrCCA1L26 in flowering time regulation
Source: BMC Genomics. 2022 May 17;23:374. doi: 10.1186/s12864-022-08620-7 (PMC9115955; doi:10.1186/s12864-022-08620-7)
Supplement: Supplementary file 4 — Additional file 4. [file 12864_2022_8620_MOESM4_ESM.pptx]

## Slide 1
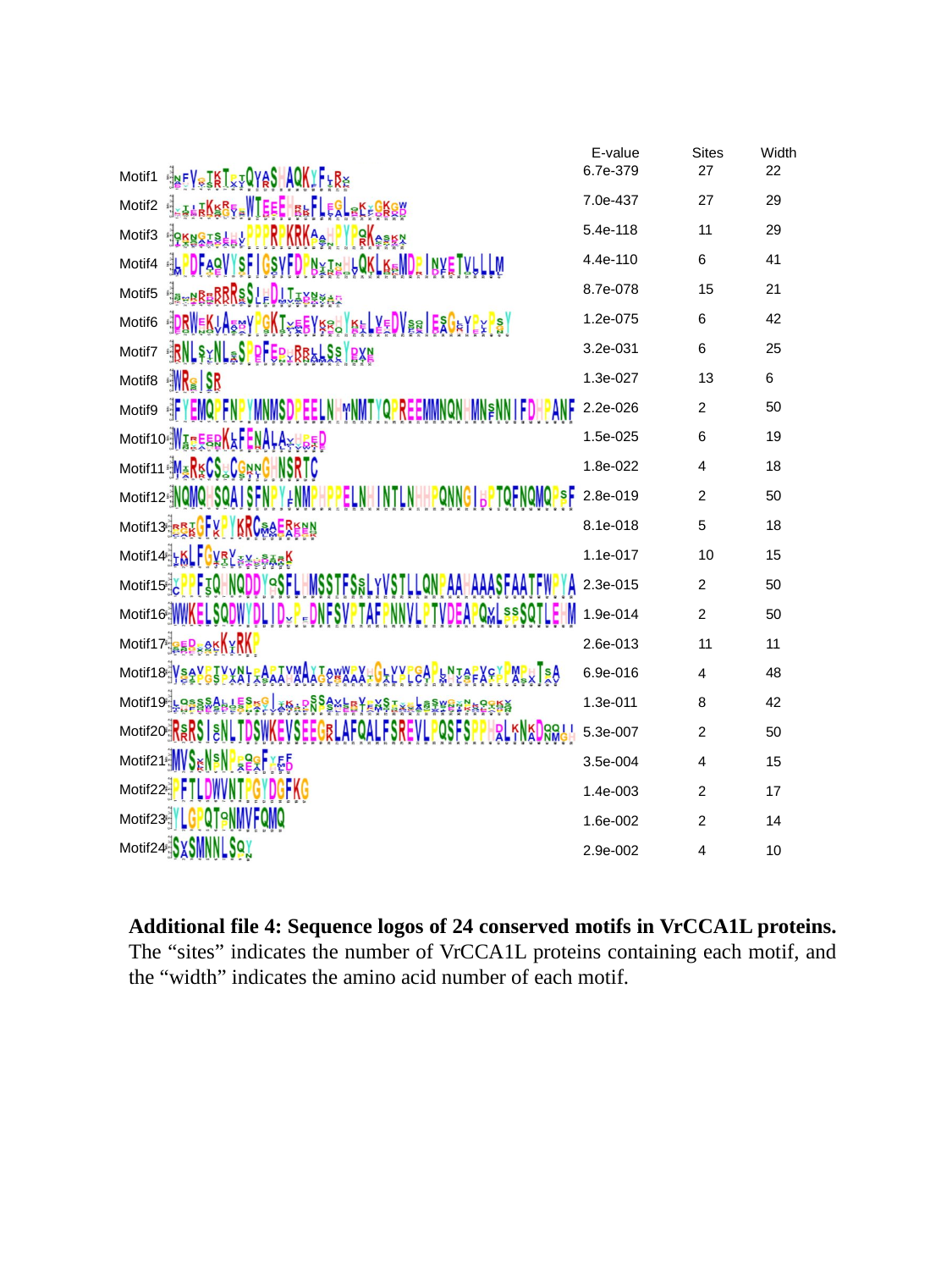

E-value
Sites
Width
6.7e-379
27
22
Motif1
Motif2
Motif3
Motif4
Motif5
Motif6
Motif7
Motif8
Motif9
Motif10
Motif11
Motif12
Motif13
Motif14
Motif15
Motif16
Motif17
Motif18
Motif19
Motif20
Motif21
Motif22
Motif23
Motif24
7.0e-437
29
27
5.4e-118
29
11
4.4e-110
41
6
8.7e-078
21
15
1.2e-075
42
6
3.2e-031
25
6
1.3e-027
6
13
50
2.2e-026
2
19
1.5e-025
6
18
1.8e-022
4
50
2.8e-019
2
18
8.1e-018
5
15
1.1e-017
10
50
2.3e-015
2
50
1.9e-014
2
11
2.6e-013
11
48
6.9e-016
4
42
1.3e-011
8
50
5.3e-007
2
3.5e-004
15
4
1.4e-003
17
2
1.6e-002
14
2
2.9e-002
4
10
Additional file 4: Sequence logos of 24 conserved motifs in VrCCA1L proteins. The “sites” indicates the number of VrCCA1L proteins containing each motif, and the “width” indicates the amino acid number of each motif.
