# Supplementary material for: Genome-wide identification and characterization of mungbean CIRCADIAN CLOCK ASSOCIATED 1 like genes reveals an important role of VrCCA1L26 in flowering time regulation
Source: BMC Genomics. 2022 May 17;23:374. doi: 10.1186/s12864-022-08620-7 (PMC9115955; doi:10.1186/s12864-022-08620-7)
Supplement: Supplementary file 6 — Additional file 6. [file 12864_2022_8620_MOESM6_ESM.pptx]

## Slide 1
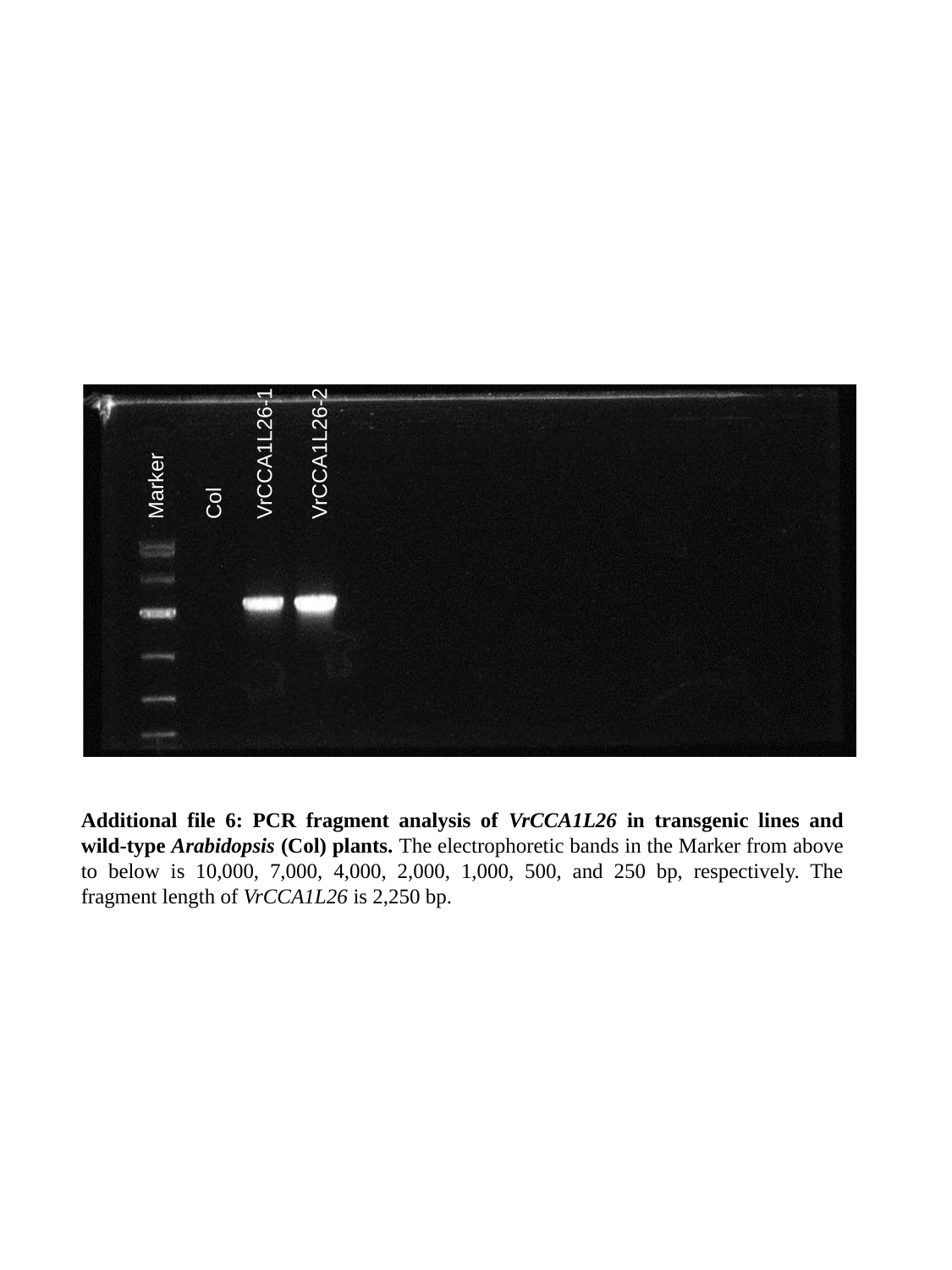

VrCCA1L26-1
VrCCA1L26-2
Marker
Col
Additional file 6: PCR fragment analysis of VrCCA1L26 in transgenic lines and wild-type Arabidopsis (Col) plants. The electrophoretic bands in the Marker from above to below is 10,000, 7,000, 4,000, 2,000, 1,000, 500, and 250 bp, respectively. The fragment length of VrCCA1L26 is 2,250 bp.
