# Supplementary material for: Genome-wide identification and characterization of mungbean CIRCADIAN CLOCK ASSOCIATED 1 like genes reveals an important role of VrCCA1L26 in flowering time regulation
Source: BMC Genomics. 2022 May 17;23:374. doi: 10.1186/s12864-022-08620-7 (PMC9115955; doi:10.1186/s12864-022-08620-7)
Supplement: Supplementary file 7 — Additional file 7. [file 12864_2022_8620_MOESM7_ESM.pptx]

## Slide 1
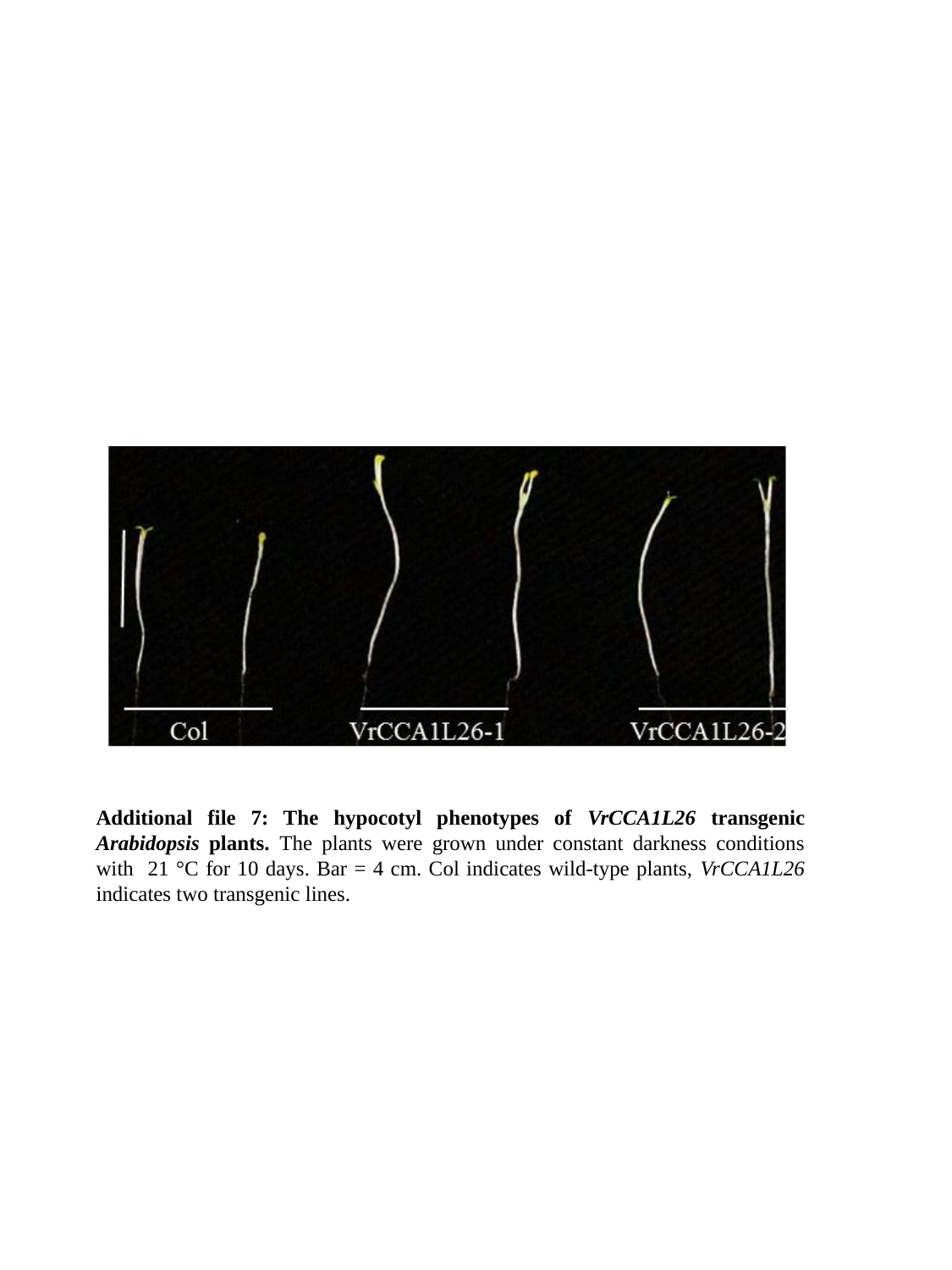

Additional file 7: The hypocotyl phenotypes of VrCCA1L26 transgenic Arabidopsis plants. The plants were grown under constant darkness conditions with 21 °C for 10 days. Bar = 4 cm. Col indicates wild-type plants, VrCCA1L26 indicates two transgenic lines.
